# A FHIR has been lit on gICS: facilitating the standardised exchange of informed consent in a large network of university medicine

**DOI:** 10.1186/s12911-022-02081-4

**Published:** 2022-12-19

**Authors:** Martin Bialke, Lars Geidel, Christopher Hampf, Arne Blumentritt, Peter Penndorf, Ronny Schuldt, Frank-Michael Moser, Stefan Lang, Patrick Werner, Sebastian Stäubert, Hauke Hund, Fady Albashiti, Jürgen Gührer, Hans-Ulrich Prokosch, Thomas Bahls, Wolfgang Hoffmann

**Affiliations:** 1 Institute for Community Medicine, Department Epidemiology of Health Care and Community Health, University Medicine Greifswald, Ellernholzstr. 1-2, 17475 Greifswald, Germany; 2 Gefyra GmbH, Otto-Hahn-Str. 9, 48161 Münster, Germany; 3 MOLIT Institute Heilbronn, Im Zukunftspark 10, 74076 Heilbronn, Germany; 4 Institute for Medical Informatics, Statistics and Epidemiology (IMISE), Leipzig University, Härtelstr. 16-18, 04107 Leipzig, Germany; 5 SMITH Consortium of the German Medical Informatics Initiative, Leipzig, Germany; 6 GECKO Institute, Heilbronn University of Applied Sciences, Max-Planck-Str. 39, 74081 Heilbronn, Germany; 7 Medical Data Integration Center (MeDIC LMU), Hospital of the Ludwig-Maximilian-University (LMU), Marchioninistr. 15, 81377 Munich, Germany; 8 Tekaris GmbH (Partner of MeDIC LMU), Elsenheimerstraße 53, 80687 Munich, Germany; 9 Chair of Medical Informatics, Friedrich-Alexander-Universität Erlangen-Nürnberg, Wetterkreuz 15, 91058 Erlangen, Germany

**Keywords:** Medical data management, Informed Consent, FHIR, Consent management, Patient rights

## Abstract

**Background:**

The Federal Ministry of Education and Research of Germany (BMBF) funds a network of university medicines (NUM) to support COVID-19 and pandemic research at national level. The “COVID-19 Data Exchange Platform” (CODEX) as part of NUM establishes a harmonised infrastructure that supports research use of COVID-19 datasets. The broad consent (BC) of the Medical Informatics Initiative (MII) is agreed by all German federal states and forms the legal base for data processing. All 34 participating university hospitals (NUM sites) work upon a harmonised infrastructural as well as legal basis for their data protection-compliant collection and transfer of their research dataset to the central CODEX platform. Each NUM site ensures that the exchanged consent information conforms to the already-balloted HL7 FHIR consent profiles and the interoperability concept of the MII Task Force “Consent Implementation” (TFCI). The Independent Trusted Third-Party (TTP) of the University Medicine Greifswald supports data protection-compliant data processing and provides the consent management solutions gICS.

**Methods:**

Based on a stakeholder dialogue a required set of FHIR-functionalities was identified and technically specified supported by official FHIR experts. Next, a “TTP-FHIR Gateway” for the HL7 FHIR-compliant exchange of consent information using gICS was implemented. A last step included external integration tests and the development of a pre-configured consent template for the BC for the NUM sites.

**Results:**

A FHIR-compliant gICS-release and a corresponding consent template for the BC were provided to all NUM sites in June 2021. All FHIR functionalities comply with the already-balloted FHIR consent profiles of the HL7 Working Group Consent Management. The consent template simplifies the technical BC rollout and the corresponding implementation of the TFCI interoperability concept at the NUM sites.

**Conclusions:**

This article shows that a HL7 FHIR-compliant and interoperable nationwide exchange of consent information could be built using of the consent management software gICS and the provided TTP-FHIR Gateway. The initial functional scope of the solution covers the requirements identified in the NUM-CODEX setting. The semantic correctness of these functionalities was validated by project-partners from the Ludwig-Maximilian University in Munich. The production rollout of the solution package to all NUM sites has started successfully.

## Background

### A national network of university medicine

The Federal Ministry of Education and Research of Germany (BMBF) is funding a national network of university medicine (NUM) to support COVID-19 and pandemic research at national level. Within the NUM project “COVID-19 Data Exchange Platform” (CODEX), as one of several funded projects of NUM, a nationwide standardised infrastructure is to be established that supports the storage and provision of COVID-19 research datasets in a data protection-compliant manner [1, 2].

For this purpose, the structured COVID-19 data of the participating 34 university hospitals (NUM sites), which include clinical data, image data and data on biospecimens [3], are to be pseudonymously transferred in the form of the German Corona Consensus data set (GECCO83) [4] in the central platform CODEX. The assessment of patient related data, the transfer to a comprehensive, standardised data repository, and the use & access procedure, that will allow researchers to analyse the data to answer urgent scientific questions, are all based on an individual informed consent.

The infrastructure required for multicentre data exchange must meet the requirements of research ethics and the EU General Data Protection Regulation (GDPR). The planned processing of a patient's health data at the NUM site and the standardised transfer to the central platform CODEX for research purposes requires, according to the GDPR Art. 6 (1) lit. a) [5], the consent of the patient.

In a complex interaction of centralised and decentralised infrastructure components, the transfer of appropriately consented health data at the NUM sites to the central platform is only indirect. Each individual site has a data integration centre (DIC) and a local Trusted Third Party (TTP) including a local consent management. The NUM sites ensure that only consented data is transferred to the central research repository CODEX. Central infrastructure components, such as the federated Trusted Third Party (fTTP), ensure, that patients can be uniquely identified across sites based on a privacy preserving record linkage (PPRL) [6]. Uniformly generated pseudonyms are provided by the fTTP. The GECCO Transfer Hub (GTH) transfers the pseudonymised research data to the central platform CODEX using the HiGHmed Data Sharing Framework (DSF) [7]. The exchange of person-identifying information (PII), health data and consent data are carried out in NUM by means of the *Health Level 7*® (HL7) standard *Fast Healthcare Interoperable Resources*® (FHIR).

### A broad consent promotes broad research

In the Medical Informatics Initiative (MII) the BMBF funded the four consortia, MIRACUM [8], HiGHmed [9], SMITH [10] and DIFUTURE [11]. The aim of the MII, which started before NUM, is, among others, “*to improve medical research and patient care with innovative data architectures and software solutions*” [12]. This project is primarily based on decentralised infrastructures and is not limited to COVID-19 or pandemic research, but shall support a very broad approach of medical research based on patient data from diagnostic and therapeutic clinical routine in all University Hospitals in Germany, and their cooperation partners.

In order to solve MII-wide challenges in a uniform manner, a number of corresponding working groups and task forces were set up. Particularly, the working group consent (WG Consent) has designed a uniform and modular consent document: the *MII Broad Consent* (version 1.6d) [13]. Moreover, the WG Consent has successfully coordinated this broad consent, which was approved by all the data protection authorities at both all federated states and the national level in Germany. It shall “*be used over all sites in the four consortia*” [12].

In close cooperation between WG Consent and the *Technology, Methods, and Infrastructure for Networked Medical Research e.V.* (TMF), the already approved *MII Broad Consent* (MII BC) [14] was extended for NUM by a NUM-specific consent module (version 1.6f) [15]. This supplementary module (called Z-module) grants patients the opportunity to decide on their own responsibility (1) whether their data may be transmitted to the central platform CODEX for the purpose of COVID-19 and pandemic research, in order to then be used for research in conformity with the GDPR, and (2) may even be transferred to countries in which the European Commission did not explicitly confirm a suitable level of data protection, yet (“unsecure third countries” [16]).

Ideally, the NUM-specific version of the MII Broad Consent (v.1.6f) is to be used at all NUM sites as a uniform basis for the data protection-compliant collection and transfer of the GECCO83 data set to the central platform CODEX.

### A harmonised representation of the patient’s consent for technical interoperability

As each site of the four MII consortia is free to choose the technical implementation for managing consent information, such as *Integrating the Healthcare Enterprise* (IHE) *Basic Patient Privacy Consents* (BPPC) or FHIR consent resources [17], the MII *Task Force Consent Implementation* (TFCI) has successfully faced the challenge of defining a technically interoperable solution [18]. The TFCI utilised globally unique object identifiers (OID) to develop a common representation of the MII Broad Consent [18].

This approach was extended with a concrete set of semantic statements for each consent module as selected by the patient (enforceable consent policies). Corresponding representations of questions, answers, modules and assigned policies of the MII Broad Consent were modelled in ART-DÉCOR [19, 20] after intensive coordination with WG Consent and TMF.

Since every NUM site is also a MII site, corresponding additions for the NUM-specific extension of the MII Broad Consent (version 1.6f) have already been considered. Despite considerable complexity, TFCI has paved the way for the technically independent exchange of semantic consent information in MII and NUM.

Each NUM site is responsible for the individual implementation of this interoperable but complex approach. This also includes ensuring that the content of the exchanged consent information is always semantically correct in order to prevent possible data protection violations (e.g. data transmissions to the central platform CODEX in the absence of a valid consent).

### A reliable consent management is essential to ensure patient’s rights

The Independent Trusted Third-Party (TTP) of the University Medicine Greifswald has successfully supported the data protection-compliance of research projects since 2014 (e.g. NAKO Health Study, German Centre for Cardiovascular Research) [21]. The open source solutions gICS® (generic Informed Consent Service), gPAS® (generic Pseudonymisation Administration Service) and E-PIX® (Enterprise Master Person Index) form the basis for the implementation of the informational separation of powers [22]. These TTP tools are part of the MIRACOLIX Toolbox 2.0 [23] of the MII consortium MIRACUM. The correct consideration and implementation of the patient's rights, in particular the handling of consents and the implementation of patient withdrawals, require a reliable consent management [12].

University Medicine Greifswald has already successfully implemented a completely digitally MII broad consent in mid of 2020 [24]. As part of the NUM-CODEX project, the University Medicine Greifswald supports the NUM sites to establish necessary local Trusted Third Parties and corresponding consent processes by providing the necessary software solution gICS for digital consent management.

Each NUM site decides individually whether to apply gICS for local consent management. Also, efforts required to integrate gICS into individual processes and infrastructures remain with the site. This typically includes not only technical aspects (e.g. network zones, authentication, various local IT-security measures), but also human resources for sufficient information of the patient, retrieval and documentation of consent, as well as for quality control of consent information.

gICS supports digital- and paper-based consent processes and follows the principles of “privacy by design” [12]. Based on consent policies and consent modules gICS allows to automatically determine and query the detailed consent status for each patient at any time [17]. The web-based functionalities of gICS have so far been provided to users exclusively via SOAP interface [25]. In the NUM-CODEX project, however, the exchange of consent information is intended solely in HL7 FHIR format. Due to a lack of uniform specification, this has not yet been satisfactorily supported by any other software solution [17].

### A standardised specification for FHIR consent

In order to be able to implement the clear objective in the NUM-CODEX project for cross-site data exchange via FHIR in the area of consents, current developments in the FHIR community must be considered. The HL7working group on consent management (WGCM), with significant participation from University Medicine Greifswald, has fundamentally renovated the handling of FHIR consent resources in collaboration with representatives from all four MII consortia. With regard to national characteristics in Germany, required profiles, vocabularies and extensions for the exchange of modular consent templates and contents were specified [26], considering applicable standards such as IHE BPPC and IHE APPC [27], and numerous examples were made available in the form of a uniform implementation guide [28]. This specification is publicly available and the balloting process started in April 2021, in order to introduce these new profiles as part of the official national HL7 FHIR standard.

The examples provided by the WGCM consider the OID-based preliminary work of the TFCI. The challenge of the syntactically and semantically correct implementation of these complex FHIR consent profiles, however, still exists and requires both extensive practical experience in the field of consent management and a comprehensive understanding of the designed FHIR consent concepts.

## Objectives

This article describes the necessary steps for the conception and implementation of a standardised solution for the FHIR-compliant provision of consent information using gICS, which can support both existing user projects and future users in the implementation of FHIR-oriented infrastructures and processes. The following conditions apply:

1. at least the FHIR-Consent Profiles as specified by the national HL7 Working Group on Consent Management (WGCM) [26] should be supported,
2. additional requirements resulting from the specific setting of NUM should be considered (such as the implementation situation at the sites, planned cross-site processes)
3. and in order to simplify the application at the sites of MII and NUM, the interoperability concept of the MII Task Force “Consent Implementation” (TFCI) based on operationalised consent policies [20] should be facilitated.

## Methods

Due to the very limited time resources in the NUM-CODEX project, the development of a solution and the coordination with the involved stakeholders, was carried out in parallel. This required intensive communication between all those involved parties and an iterative approach adapted to the particular situation.

### Extending the modular TTP approach

Whereas the separate TTP tools have been used for each area of responsibility of current Trusted Third-Party implementations [21], a distinct module should be applied for consistent FHIR format conversions. This particular “Trusted Third-Party FHIR Gateway” (TTP-FHIR Gateway) should serve as an intermediary between external FHIR-specific infrastructure components and existing TTP tools like gICS. This approach has two advantages:

1. As a stand-alone software component, an integration of the TTP-FHIR Gateway into existing infrastructures can be achieved with little effort: existing application servers that already provide TTP services based on the TTP tools are simply supplemented with another deployment.
2. An implementation in different application scenarios is conceptually possible by an appropriately configurable TTP-FHIR Gateway and without additional efforts for (a) sites which use gICS only as well as (b) sites which use a combination of TTP tools like gICS, gPAS, E-PIX or TTP dispatcher [21].

### Identifying a basic set of FHIR-functionalities based on stakeholder dialogue

The TTP-FHIR Gateway for gICS shall be used at the individual NUM sites and thus support the planned data flows from 34 NUM sites via the GECCO Transfer Hub (GTH) to the central platform CODEX. For this purpose, options for simplification of connecting the sites to the NUM infrastructure were discussed with partners from the University Medicine Erlangen, including queries to the local consent management via TTP-FHIR Gateway.

The partner site Heilbronn is responsible for the implementation of the GTH using the DSF [7]. The FHIR consent resources delivered by the TTP-FHIR Gateway were consolidated with the partner Heilbronn, as the GTH ensures that only correctly consented GECCO data sets may be transmitted to the central platform. This validation is currently planned for implementation based on the TFCI concept for interoperability.

In order to support these planned processes correctly, four essential functionalities for the TTP-FHIR Gateway to handle FHIR consents resources were identified from this stakeholder dialogue:

1. Retrieval of all consents of a specific research project (consent domain)
2. Retrieval of the most current consent of a specific patient (regarding a specified consent template)
3. Patient-specific check of the consent status (e.g. “*Is the scientific use of the patient's medical data for the intended purpose legally permissible at the current time?*”)
4. Retrieval of a complete set of documented consent policies signed by a patient classified by “deny” or “permit” [29]

According to the interface specification, gICS 2.12.0 covers these functionalities via the freely available and documented SOAP interface [30]. The FHIR-compliant specification of corresponding FHIR functionalities, including necessary request and response parameters, had to be added.

### Ensuring a FHIR-compliant design

In order to ensure the syntactic correctness of FHIR-related contents and functionalities, the necessary FHIR resources and FHIR operations were profiled and extended in close cooperation with experts from Gefyra [31], who are actively involved in the German FHIR Technical Committee and provide comprehensive FHIR consultancy and training. Supported by Gefyra, numerous profiles, code systems, operation definitions and search parameters were publicly specified [32]. An implementation guide with additional explanations and relevant examples [33] was drafted in parallel.

### Expanding gICS and implementing the TTP-FHIR Gateway

gICS is the data-holding system for all consent-relevant information in the outlined application scenario. This includes the reference to the research project (consent domain), the reference to the consenting person, applied consent templates, provided signatures and documented scans. Within a FHIR server, managed resources are always uniquely referenced via corresponding Universally Unique Identifiers (UUID). To provide technically valid FHIR resources via TTP-FHIR Gateway, gICS was enabled to generate these required FHIR UUIDs.

The TTP-FHIR Gateway has been implemented using the FHIR HAPI (version 5.4.0) [34]. FHIR-compatible functionalities are provided for client-side use using a gICS-related FHIR endpoint (base-URL: *http[s]://<host>:<port>/ttp-fhir/fhir/gics*).

The implementation comprehensively considers the profiles specified and balloted by the WGCM [26]. At the same time, it includes selected gICS-relevant extensions, for example for the implementation of consent policies in FHIR [35] (these were deliberately left out of the work of the WGCM) and for the complete mapping of all necessary information for consent templates [36] (among other things to map additional labels and properties).

In addition, several mechanisms for the customisation of FHIR consent resources, in terms of value codings and answer options, were integrated into the TTP-FHIR Gateway. This will be particularly relevant for application at NUM and MII sites.

### Supporting NUM sites in the implementation

As part of the MII, University Medicine Greifswald has already prepared uniform digital consent templates in the form of a gICS-specific import format [17] based on the consent documents developed by TMF and AG Consent (Broad Consent, version 1.6.d) and made them available to all MII sites for free use and individualisation.

This consent-template was extended according to the content requirements of the NUM-specific Broad Consent (version 1.6.f) for the data protection-compliant collection and transfer of the GECCO83 data set to the central platform CODEX. Numerous consent policies were assigned to the individual consent modules of the template, as provided in the concepts of the TFCI [20].

The consent-template was provided with so-called “*Expiration Properties”* [37] in order to consider the time constraints provided by the MII (including validity of consent for 30 years, permissibility of medical data collection for 5 years). These are evaluated in real time by gICS when valid consents are requested.

Furthermore, the consent-template has been annotated with numerous “*External Properties”* [37] to inject FHIR-relevant information. This additional information is considered in the subsequent derivation of FHIR consent resources with TTP-FHIR Gateway and ensures technical conformity with the specifications of the TFCI (e.g. specifications for OIDs and permissible answer options) (cf. examples of FHIR consent resources in the Implementation Guide of the WGCM [38]).

### Assuring semantic quality of the implemented FHIR functionalities

Pre-releases of the new and extended gICS, the TTP-FHIR gateway and the created consent template were handed over early for external evaluation to the NUM-CODEX project partner, the Medical Data Integration Center of clinical centre of the Ludwig Maximilian University of Munich (MeDIC^LMU^). MeDIC^LMU^ conducted initial integration tests using Apache JMeter. The focus of the tests was on ensuring the semantic correctness of the FHIR consent resources generated by the TTP-FHIR Gateway.

## Results

### gICS is “on FHIR”

**Fig. 1:**
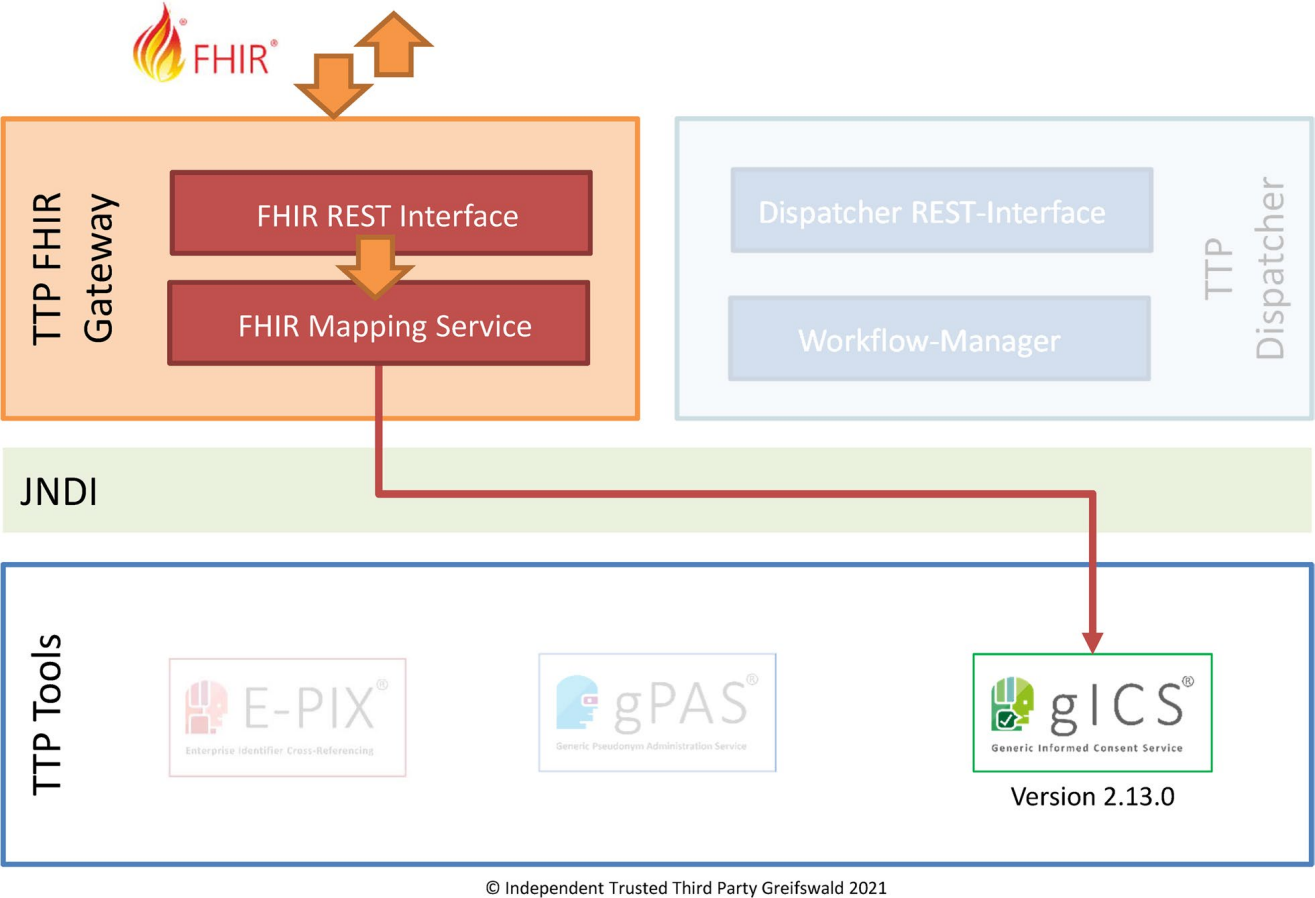
The modular architecture of the TTP-FHIR Gateway supports simplified provision of consent-related content from the consent management gICS as FHIR resources (The authors have the necessary rights to use the registered image trademarks of E-PIX, gPAS and gICS)

The TTP-FHIR Gateway provides a FHIR interface with selected search parameters and FHIR operations. It allows a FHIR-compliant provision of consent information, which is primarily managed by gICS, as FHIR resources. For the time being, the TTP-FHIR Gateway acts exclusively as a mapping service between FHIR calls of the requesting system and the managed functionalities of gICS (cf. Fig. 1). The necessary communication is implemented within the application server via Java Naming and Directory Interface (JNDI). The TTP-FHIR Gateway is an independent software component and relies on gICS application logic. The evaluation of the provided FHIR contents is the responsibility of the requesting system (e.g. local infrastructure component at the specific NUM site).

The TTP-FHIR Gateway can be integrated into existing infrastructures with little effort. Existing application servers that already deploy a selection of TTP tools (e.g. gICS, E-PIX, gPAS or the TTP dispatcher) are simply supplemented by another deployment. After successful deployment, a gICS-specific FHIR endpoint is available for use under the specified base-URL. Securing the new FHIR interface by means of the established Single-Sign-On solution (SSO) Keycloak [39] is supported. The generic Informed Consent Management Service gICS (version 2.13.0) by default includes the implemented TTP-FHIR Gateway. It is available for download [40] in Docker-Compose-format for all NUM sites and other interested users.

### FHIR functionalities comply with the new official FHIR consent profiles

The TTP-FHIR Gateway for gICS provides access to gICS consent content in accordance with the new and officially balloted FHIR consent profiles of the WGCM [26]. The functionalities identified according to the stakeholder dialogue were implemented as FHIR operations and are summarised in Table 1.

**Table 1:** Overview of the FHIR operations supported in the first release of the TTP-FHIR Gateway for gICS (version 2.13.0)

Purpose	Invocation	Specification and examples
Retrieval of all consents of a specific research project (consent domain)	$allConsentsForDomain	https://simplifier.net/guide/ttp-fhir-gateway-ig/allConsentsForDomain
Retrieval of the most current consent of a specific patient with regards to a specific consent template	$currentConsentForPersonAndTemplate	https://simplifier.net/guide/ttp-fhir-gateway-ig/currentConsentForPersonAndTemplate
Patient-specific check of the consent status for a specific consent policy	$isConsented	https://simplifier.net/guide/ttp-fhir-gateway-ig/isConsented
Retrieval of a complete set of documented consent policies signed by a patient	$policyStatesForPerson	https://simplifier.net/guide/ttp-fhir-gateway-ig/policyStatesForPerson

Further technical details are explained in the German Implementation Guide [33]

With the specifications and examples made publicly available early on, interested partners and NUM sites were able to gain early insight into the progress of implementation [33] and adapt their local consent processes. The FHIR profiles of the WGCM form the formal basis of the FHIR consent exports. Additional comprehensive search parameters were defined for the gICS implementation [41]. Among other things, the characteristics of the FHIR exports for selected resource types (e.g. consent domains, consent templates and consent modules) can be controlled by specifying the *_profile* parameter. Thus, respective FHIR resources can be provided according to the specifications of the WGCM, but also in an extended form according to the extended FHIR profiles for gICS.

A comprehensive specification of all implemented profiles, supported FHIR search parameters and gICS-related extension profiles, required operations, considered code systems and defined value sets is publicly provided in the implementation guide [33] of the corresponding Simplifier-project curated by the *Independent Trusted Third Party of the University Medicine Greifswald* and *Gefyra* [32].

### A customised consent template simplifies assurance of technical interoperability

**Fig. 2:**
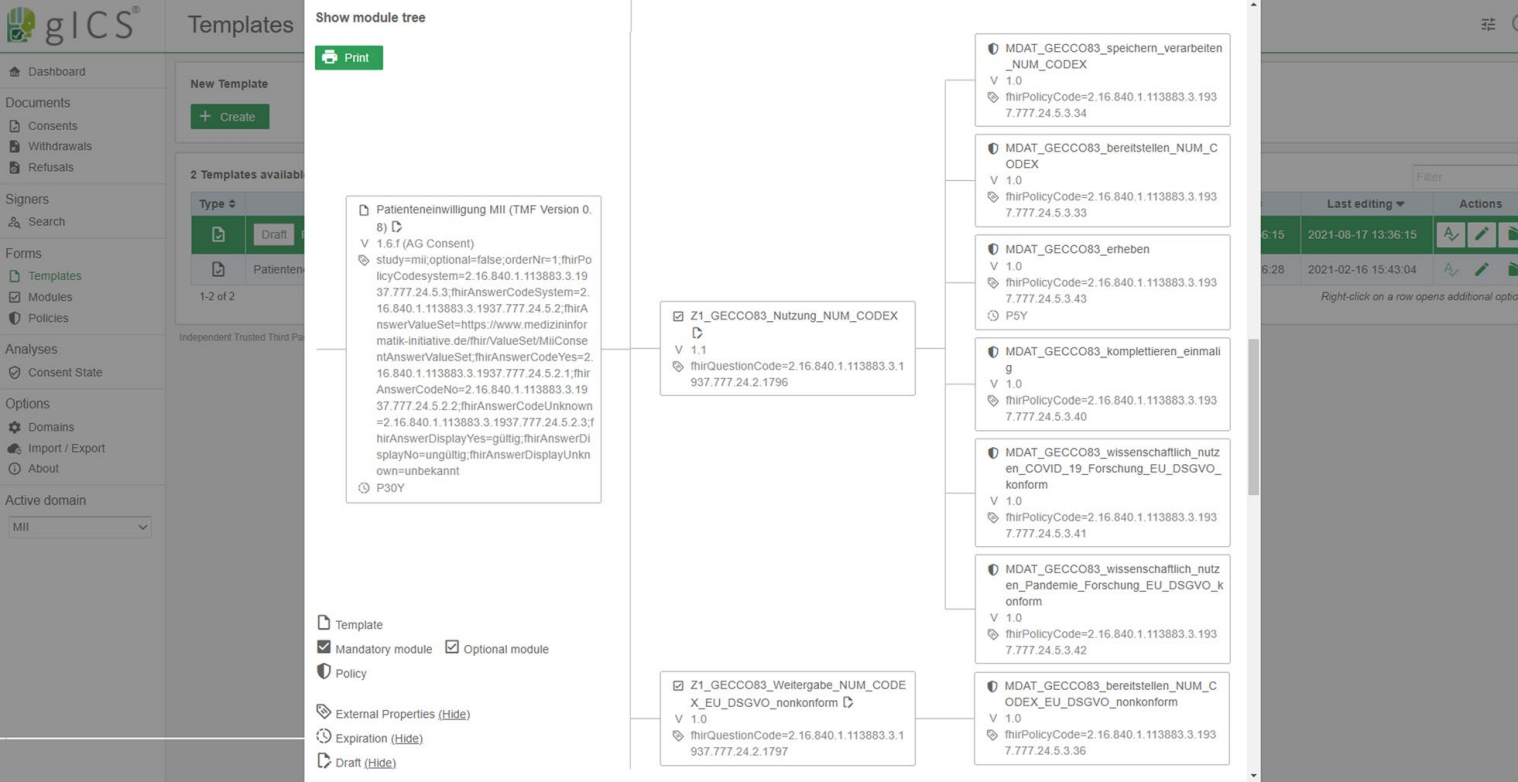
Structural representation of the provided consent template (detailed view) with TFCI-specific external properties. These are evaluated by the TTP-FHIR Gateway and considered when generating FHIR resources (The authors have the necessary rights to use the registered image trademarks of E-PIX, gPAS and gICS)

For all NUM and MII sites, a modular consent template (cf. Fig. 2) has been provided as a supplement to the gICS release [42, 43]. This directly importable template not only implements the text specifications of the Broad Consent (version 1.6.f) [15] as consolidated by the TMF and WG Consent, but also fully considers the interoperability concept developed by the TFCI [20], which is based on globally unique object identifiers (OID) for each consent policy.

As soon as consents based on this template are managed in gICS, these consent contents can be exported to FHIR consent resources via the TTP-FHIR Gateway. By default, gICS-specific references and codings are used for e.g. answer options in templates (FHIR questionnaires [28]), references to specific questions in consent modules (FHIR template frame [28]), and coding for policy semantics (FHIR consent.provisions [38]).

When generating FHIR resources, individualisation markers (external properties) contained within the prepared MII- and NUM-template are evaluated and interpreted by the TTP-FHIR Gateway. In this way, the customisation of FHIR consent resources could be successfully implemented according to the concepts of the TFCI and with respect to examples provided by the WGCM [28]. This includes the use of:

1. policy OIDs in FHIR consent resources (FHIR consent.provision.coding),
2. question OIDs in FHIR questionnaire resources (FHIR questionnaire.item.linkId), as well as
3. response options (FHIR questionnaires) and responses (FHIR questionnaire response [28]).

The advantage of this approach is that future changes to these code systems and value sets can be addressed without changing program code and only by updating the consent template. The corresponding manual presents a comprehensive overview of the supported external properties and their impact on generated FHIR representations [37].

Comprehensive examples of FHIR consent resources generated by the TTP-FHIR Gateway, based on the provided template and including necessary references to the TFCI-concept (as part of FHIR consent.provision) are available from Simplifier [44] accompanied by detailed descriptions within the Implementation Guide [33].

### External partners approved semantic quality of FHIR functionalities

Based on the information and materials provided by the University Medicine Greifswald (gICS, TTP-FHIR Gateway, consent template, documentation), MeDIC^LMU^ has developed and executed test cases to validate the correctness of the generated FHIR-based consent (related to the functional scopes shown in Table 1), as well as the conformity with expectations related to request-parameters and response-error-codes as provided by the TTP-FHIR Gateway.

In summary, MeDIC^LMU^ as an independent project partner, has performed software test covering the semantic correctness (according to existing specifications [28, 33]) of the provided FHIR operations and resources through a total of 28 automated test cases (which are available upon request from the corresponding author). The test environment included the gICS 2.13.0 and TTP FHIR Gateway 2.0.0 (Snapshot Version 2021-06-21), as well as Apache JMeter 5.4.1. The defined test cases were designed and run based on provided sample data and MII BC template (1.6.f incl. Z-Module (TMF V0.8)).

In addition, inconsistent/missing http-error codes for return values were identified in 3 of the 28 test cases (10.71% of the test cases). Considering these results, the University Medicine Greifswald corrected the corresponding implementation of the TTP-FHIR Gateway and updated the documentation in the Implementation Guide accordingly.

The NUM and MII sites were centrally provided with the approved version of gICS with FHIR support (gICS version 2.13.0 with TTP-FHIR Gateway) [40], as well as a TFCI-compliant Broad Consent templates (1.6d [42] and 1.6.f [43]) on June 29th, 2021.

### Rollout at the NUM sites started

The implementation of consent management at the 34 NUM sites is continuously monitored on the project´s collaboration platform [45]. As of August 20th, 2021, 17 sites currently intend to use gICS (installation successful: 10; installation in progress: 7). 10 of these sites have already imported the template provided (NUM-template with Z-module: 8; MII-template: 2). In total, 7 NUM and MII sites are already using gICS and the template productively.

## Discussion

The technical implementation discussed here represents the first full implementation of the FHIR profiles of the WGCM in an open-source solution for digital consent management. These FHIR consent profiles were balloted in April 2021 and became part of the official FHIR standard (at national level in Germany) in September 2021 [46].

The FHIR implementation provided is freely available as a combination of gICS and TTP-FHIR Gateway since the end of June 2021 [40]. It represents a milestone for the NUM and MII sites to achieve the set goals of exchanging consent content in FHIR format. Due to the limited time and personnel resources in NUM, functionalities were initially prioritised and only basic functionalities were implemented. These functionalities will be iteratively extended in subsequent releases, taking user feedback into account.

The developed solution (gICS with TTP-FHIR Gateway-Extension) is explicitly not limited to an application in the context of MII and NUM. Existing gICS user projects, such as the German National Cohort (NAKO) [47] and the German Centre for Cardiovascular Research (DZHK) [48], can also export existing consents, consent templates, modules and policies in valid and tested FHIR format by simply updating to the latest version of gICS [49].

The provided consent template (MII BC 1.6.f) encapsulates the necessary prior technical knowledge to generate FHIR consent resources conforming to the specifications of the TFCI concepts and to consider them consistently and uniformly. The provision of gICS, TTP-FHIR Gateway and the consent-template are an offer to the NUM and MII sites. Their use simplifies and supports the uniform exchange of health data between the NUM sites and the central research platform in conformance with the patients’ consents. It is up to each site to accept this offer and to integrate these tools into local Trusted Third-Party processes and, thus, reduce necessary technical efforts for a HL7 FHIR- and TFCI-compliant consent management.

Nevertheless, this requires obtaining the corresponding ethics approval of the local ethics as well as necessary local processes for informing the patient, obtaining and documenting consent and realisation of corresponding withdrawal processes, which is also complex and individual for each site.

## Conclusions

In late 2020, the NUM and MII sites have made a binding commitment to set up local infrastructures and establish required local consent processes to enable a data protection-compliant export of GECCO83 data items (based on HL7 FHIR) to the central research platform CODEX to support research on COVID-19 and to demonstrate the necessary degree of preparedness for future pandemic situations in Germany.

In the first funding phase of NUM-CODEX, a FHIR-based representation of informed consent, based on the MII Broad Consent (MII BC), signed by the patient and automatically enforceable, was to be transferred in addition to the patient’s medical data. This should not only comply with the new HL7 FHIR consent profiles [28], but also with the interoperable implementation concept of the TFCI [20].

In order to support the 34 NUM sites in this challenge, this article has shown that the technical prerequisites for a FHIR-compliant and interoperable provision of consent information could be achieved with the help of the consent management gICS and the provided TTP-FHIR Gateway [40].

The successfully implemented TTP-FHIR Gateway for gICS enables the FHIR compliant provision of consent information. This solution can be used for existing and future user projects of gICS. It can as well support the implementation of FHIR-oriented infrastructures and data-protection-compliant processes. The TTP-FHIR Gateway supports the balloted FHIR consent profiles of the HL7 Working Group Consent Management [28]. All gICS-specific extensions were specified and publicly documented with the support of FHIR experts [31]. The initial functional scope of the TTP-FHIR Gateway coincides with the requirements identified in the NUM-CODEX setting. The semantic correctness of these functionalities was tested by externally conducted tests at the clinical centre of the Ludwig-Maximilian University Munich. In order to simplify the technical application of the MII BC and the corresponding implementation of the TFCI interoperability concept at the NUM sites, a pre-configured consent template was created in accordance with specifications of the MII BC [13, 15] and was provided to all NUM partner sites [42, 43].

In the NUM-CODEX project, the interaction of gICS and HL7 FHIR in terms of content and technology was successfully demonstrated using the example of the MII BC. The implementation approach explained can be used for the currently known variants of the MII BC. Moreover, this approach can easily be customised for application in additional scenarios out of the MII BC.

This is particularly advantageous, if it should not be possible to establish the MII BC at selected university hospitals due to ethical or legal concerns. In this case, alternative options for the export of pseudonymised data to NUM-CODEX could be considered. (e.g. use of research data from other local COVID-19 projects collected on the legal basis of individual consent documents). The clarification of the permissibility of this procedure would, of course, primarily be a legal-ethical question. A technical implementation can only be considered, if the informational self-determination of the patient is not impaired.

## Data Availability

The consent management software (gICS 2.13.0 with TTP-FHIR Gateway) [40], the consent templates developed for documentation of MII- and NUM-CODEX consents [42, 43] and the referenced FHIR Consent profiles [46] are publicly available.

## Abbreviations

APPC: Advanced Patient Privacy Consent
BPPC: Basic Patient Privacy Consents
CODEX: COVID-19 Data Exchange Platform
DSF: Data Sharing Framework
FHIR®: Fast Healthcare Interoperable Resources
GDPR: EU General Data Protection Regulation
gICS®: Generic Informed Consent Service
GTH: Gecco Transfer Hub
HL7®: Health Level Seven International
IHE: Integrating the Healthcare Enterprise
JNDI: Java Naming and Directory Interface
MeDIC^LMU^: Medical Data Integration Center of the clinical centre of the Ludwig Maximilian University of Munich
MII: Medical Informatics Initiative
MII BC: MII Broad Consent
MIRACUM: Medical Informatics in Research and Care in University Medicine (MI-I Consortium)
NUM: Network of university medicine (NUM) to support COVID and pandemic research at national level in Germany
TFCI: MII Task Force Consent Implementation
TMF: Technology, Methods, and Infrastructure for Networked Medical Research e.V. (Technologie- und Methodenplattform für die vernetzte medizinische Forschung e.V.)
TTP: Trusted Third Party
UMG: University Medicine Greifswald
WG Consent: MII Working Group Consent
WGCM: Working Group Consent Management
